# The relationship between sleep disturbances, depression and anxiety in differentiated thyroid cancer patients during radioiodine (131I) therapy: a longitudinal observational study

**DOI:** 10.3389/fendo.2026.1743676

**Published:** 2026-02-02

**Authors:** Wanmin Huang, Yusheng Zheng, Jinyan Guo, Chunliu Luo

**Affiliations:** 1 Department of Oncology, The Affiliated Panyu Central Hospital, Guangzhou Medical University, Guangzhou, Guandong, China; 2 Interventional Center, Maoming People's Hospital, Maoming, Guandong, China; 3 Department of Clinical Nutrition, The Affiliated Panyu Central Hospital, Guangzhou Medical University, Guangzhou, Guandong, China; 4 Medical Imaging Center, The First Affiliated Hospital, Jinan University, Guangzhou, Guandong, China

**Keywords:** 131I therapy, anxiety, depression, sleep disturbances, thyroid cancer

## Abstract

**Background:**

The temporal dynamics of sleep disturbances following radioactive iodine (RAI) therapy for differentiated thyroid cancer (DTC), along with their underlying mechanisms and associated factors, remain poorly understood. This gap underscores the need for longitudinal investigations.

**Methods:**

We conducted a longitudinal study of 160 DTC patients from Guangdong Southern Teaching Hospital. Self-assessment scales were used to assess patients’ knowledge of the purpose of ¹³¹I therapy, depression, anxiety symptom and sleep assessment scales were used to assess with the Pittsburgh Sleep Quality Index, Insomnia Severity Index, Self-Rating Depression Scale (SDS) and Self-Rating Anxiety Scale (SAS). Assessments were conducted at three time points: pre-therapy, peri-therapy (during hospitalization), and post-therapy.

**Results:**

Of the 160 patients, 148 completed the study (53 males [35.8%]; 95 females [64.2%]). Risk factors associated with sleep disorders included anxiety, snoring, educational level, and work/study-related stress. Longitudinal trajectory analysis revealed nonlinear trends: the incidence of sleep disorders (35.8% → 52.9% → 32.6%), anxiety symptoms (23.6% → 40.7% → 14.0%), and depressive symptoms (47.3% → 56.4% → 33.3%) all increased initially before declining.

**Conclusion:**

With increasing treatment duration, the incidence of sleep disorders, anxiety, and depression initially rose and subsequently declined. Sleep quality reached its lowest point at the end of treatment, whereas anxiety and depression levels peaked one month post-therapy before decreasing to levels below the pre-treatment baseline.

## 1 Introduction

Thyroid cancer (TC), the most common endocrine malignancy, exhibits a global age-standardized incidence rate of 10.1 per 100,000 in women and 3.1 per 100,000 in men (1). Among TC cases, differentiated thyroid cancer (DTC) is the predominant subtype, typically managed through a multimodal approach that includes surgical resection, adjuvant radioactive iodine (RAI) therapy, and thyroid-stimulating hormone (TSH) suppression therapy (2). Postoperative RAI ablation of residual thyroid tissue is a cornerstone of DTC management, as it effectively targets thyroid cells while sparing surrounding healthy tissues (3). This precision is achieved through the unique ability of thyroid cells to selectively absorb iodine, enabling RAI to deliver highly accurate and targeted therapy (4).

The therapeutic effect of RAI is primarily mediated by β-particles, which have a short tissue penetration range of 0.8–2 mm, enabling precise ablation of thyroid follicular cells. However, the emitted γ-rays, due to their higher penetration capability, may pose a radiation exposure risk to surrounding individuals (5). In addition to its therapeutic benefits, RAI therapy is associated with various adverse effects, including bone marrow suppression, gastrointestinal disturbances (particularly nausea), cervical discomfort, and dysfunction of the lacrimal and salivary glands (6). Furthermore, patients undergoing RAI therapy may potentially expose their family members to radiation through multiple pathways, such as the excretion of radioactive substances in bodily fluids (e.g., saliva and urine) and direct external radiation emission from their bodies (7). Consequently, strict radiation protection protocols are implemented during RAI administration, requiring patients to limit physical activity and observe social isolation measures to minimize radiation exposure to themselves and others.

However, patients often demonstrate limited comprehension regarding the potential complications associated with RAI therapy. This insufficient understanding frequently contributes to the development of significant psychological distress, manifesting as anxiety, fear, and depressive symptoms (8). According to a recent study by Su et al. (2023), a significant proportion of DTC patients exhibited psychological distress following RAI therapy, with 48.61% demonstrating clinically significant anxiety symptoms and 47.22% showing depressive symptoms at the one-month follow-up (9). These psychological complications, compounded by the physical side effects of RAI therapy, may significantly impair patients’ sleep quality and overall quality of life (8).

Several studies have further investigated the impact of RAI therapy on sleep quality and psychological morbidity. For example, a case-control study by Afrashteh et al. (2022) demonstrated that patients with thyroid cancer, including those treated with RAI, experienced significantly poorer sleep quality and higher stress levels compared to healthy controls (10). Additionally, a prospective longitudinal study by Koo et al. (2022) followed papillary thyroid carcinoma patients for five years and found that sleep quality declined significantly following RAI therapy, with persistent disturbances observed during long-term follow-up (11). However, these studies are limited by their focus on either cross-sectional comparisons or long-term outcomes without detailed assessment of the immediate and short-term effects of RAI therapy. Furthermore, the underlying mechanisms and temporal patterns of these adverse effects remain poorly understood.

A cross-sectional study by Gao et al. (2022) highlighted the high prevalence of psychological distress among thyroid cancer patients during the transitional period of treatment, emphasizing the need for targeted interventions to address these issues (12). Similarly, Giordano et al. (2024) conducted a comprehensive assessment of psychological status in thyroid cancer patients undergoing radionuclide therapy, revealing significant anxiety and depressive symptoms during the early phases of treatment (13). Moreover, sleep disorders are influenced by several factors,including snoring, educational level and smoking history et al (14). Lin et al. (2024) identified several factors associated with psychological distress in thyroid cancer patients, including treatment-related side effects and social isolation, further underscoring the complexity of these adverse effects (15). However, it is unclear how these differences may impact DTC patients sleep quality that cause long-term impacts for their quality of life.

Despite these findings, the temporal dynamics and underlying mechanisms linking RAI therapy to psychological morbidity remain poorly characterized, highlighting the need for longitudinal studies to address these gaps. To address these gaps, we hypothesize that RAI therapy significantly affects sleep quality and psychological well-being in DTC patients, with distinct temporal patterns observable before and after treatment. This study aims to prospectively assess these changes through a longitudinal observational design, providing critical insights for developing targeted interventions to optimize patient care and therapeutic outcomes.

## 2 Materials and methods

### 2.1 Study design and settings

This longitudinal observational study examined sleep quality and mental health trajectories in patients with differentiated thyroid cancer (DTC) receiving postoperative radioactive iodine (¹³¹I) therapy. Participants were consecutively recruited from the Nuclear Medicine Department of a tertiary hospital in Guangzhou, China, between December 2020 and December 2021. Among 160 questionnaires distributed at baseline (T_0_), 148 valid responses were collected (response rate: 92.5%). Longitudinal data were collected at three stages: T_0_ (Baseline, 2 weeks before ¹³¹I therapy), T_1_ (Post-treatment, 24 hours after therapy completion), and T_2_ (Follow-up, 1 month post-discharge).

Patients should stop thyroid hormone medication 3–4 weeks before undergoing 2-week comprehensive examinations. The first survey assesses baseline levels of sleep, anxiety, and depression in pre-booked individuals. In the nuclear medicine ward, the hospital stay lasts 24–48 hours, with the second survey conducted 24 hours post-treatment to evaluate changes in sleep and psychological state. After iodine therapy, patients resume thyroid hormone intake, with hormone levels typically returning to normal one month post-treatment. However, patients are advised to avoid close contact with children for one month post-discharge to minimize public radiation exposure, with social activities still restricted. The third data collection occurs during the follow-up visit one month after discharge. All patient information is recorded by three trained nurses, who notify follow-up times and ensure the continuity of data collection and patient compliance.

### 2.2 Sample size calculation

Based on multivariate correlation studies, the sample size is calculated using the formula N= (U_α_S/δ) ^2^. With a significance level of α=0.05, the corresponding Uα is 1.96. The allowable error δ is set between 0.1 and 0.2S. This calculation results in a required sample size N ranging from 96 to 384. Accounting for a dropout rate of approximately 10%, the adjusted sample size should be between 105 and 422.

### 2.3 Assessment tools

Sociodemographic characteristics were assessed using a structured questionnaire developed through literature review and self-assessment scale were used to assess patients’ knowledge of the purpose of ¹³¹I therapy, the environment, and the amount of radiation. Sleep quality was evaluated with the Pittsburgh Sleep Quality Index (PSQI), and Insomnia Severity Index (ISI); psychological status was measured using the Self-Rating Depression Scale (SDS) and Self-Rating Anxiety Scale (SAS).

#### 2.3.1 Pittsburgh sleep quality index

The Pittsburgh Sleep Quality Index (PSQI) is a validated 19-item instrument assessing subjective sleep quality across seven domains: sleep latency, duration, efficiency, disturbances, medication use, daytime dysfunction, and perceived sleep adequacy. Each domain is scored 0-3, with global scores ranging from 0 (optimal) to 21 (severely impaired). A cutoff score >5 discriminates poor sleepers (sensitivity: 90-99%; specificity: 84-87%) (16). In this cohort, the scale demonstrated good internal consistency (Cronbach’s α=0.842).

#### 2.3.2 Insomnia severity index

The Insomnia Severity Index (ISI) is a validated 7-item instrument assessing insomnia severity over the preceding two weeks. Each item employs a 5-point Likert scale (0=none to 4=very severe), yielding total scores from 0-28. Interpretation thresholds are: 0-7 (no clinically significant insomnia), 8-14 (subthreshold insomnia), 15-21 (moderate clinical insomnia), and 22-28 (severe clinical insomnia) (17). The scale demonstrated acceptable internal consistency in this sample (Cronbach’s α=0.804).

#### 2.3.3 Self-rating depression scale

The SDS is a validated 20-item self-administered instrument assessing depressive symptomatology. Items are rated on a 4-point Likert scale (1=rarely to 4=almost always). Raw total scores (range: 20-80) are converted to a standardized metric by multiplying by 1.25. Standardized scores ≥50 (equivalent to raw score ≥40) indicate clinically significant depression, with severity categorized as: <50 (none), 50-59 (mild), 60-69 (moderate), and ≥70 (severe) (18). Internal consistency was excellent in this cohort (Cronbach’s α=0.880).

#### 2.3.4 Self-rating anxiety scale

The SAS is a psychometrically robust 20-item measure of anxiety severity. Utilizing a 4-point Likert-type scale (total raw score range: 20-80), standardized scores are derived by multiplying raw totals by 1.25. Clinical anxiety is defined as standardized scores ≥50, stratified as: 50-59 (mild), 60-69 (moderate), and ≥70 (severe) (19). Higher scores indicate greater anxiety severity. The scale showed strong reliability in this sample (Cronbach’s α=0.880).

### 2.4 Statistical analysis

Sociodemographic and clinical characteristics of the comparative groups were summarized using descriptive statistics (means ± SDs, medians with interquartile ranges). Categorical variables were analyzed through χ² tests. Longitudinal changes in sleep quality, anxiety, and depression scores were evaluated using repeated-measures ANOVA. Partial correlation analyses explored pairwise associations between these domains while adjusting for covariates. Binary logistic regression models identified sleep disorder risk factors incorporating demographic and clinical covariates. All analyses were conducted in SPSS (v26.0) with α=0.05 defining statistical significance.

## 3 Results

### 3.1 Demographic and clinical characteristics

**Figure 1:**
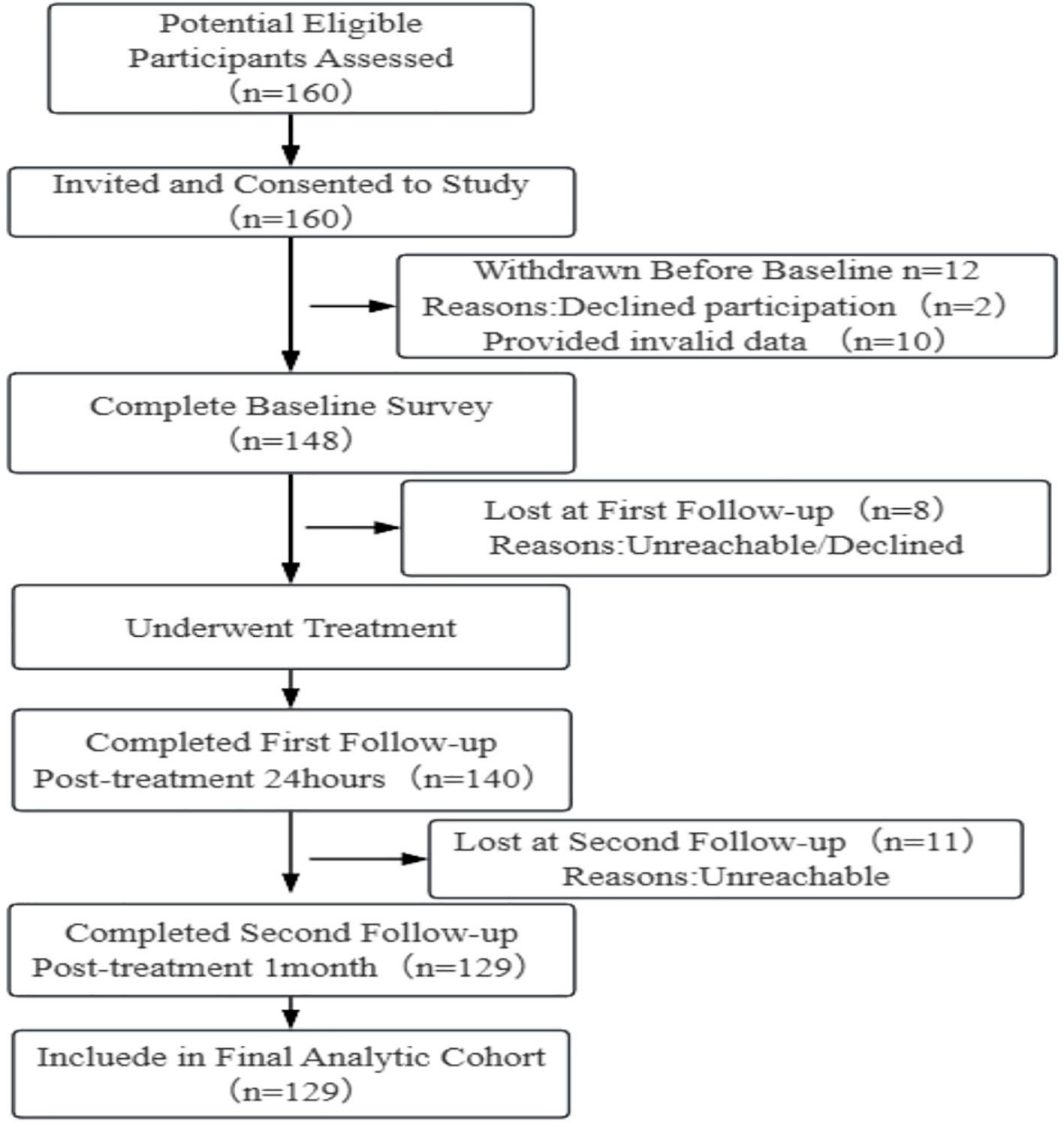
Flowchart of the study.

A total of 148 participants were enrolled in this study. After ^131^I therapy, a total of 140 cases were investigated, with 2 cases refusing to participate and 6 cases with invalid questionnaires. After one month of ^131^I therapy, a total of 129 cases were investigated, with 11 cases lost to follow-up (**Figure 1**). The lost to follow-up rates for the two occasions were 5.4% and 7.9% respectively. The age range was 14–65 years old and the mean age was 37.09 ± 11.08. Other demographic data are shown in **Table 1**.

**Table 1:** Demographic of the DTC patients (n=148).

Characteristics		n	%
Sex	Male	53	35.8%
	Female	95	64.2%
Age (years)	≤20	4	2.7%
	21-35	77	52.0%
	36-50	46	31.1%
	≥51	21	14.2%
Education	Technical secondary school and below	53	35.8%
	Junior college student	33	22.3%
	Undergraduate	47	31.7%
	Postgraduate degree	15	10.2%
Work interests	High	63	42.6%
	Moderate	81	54.7%
	Low	4	2.7%
Work stress	Low	76	51.3%
	Moderate	70	47.3%
	High	2	1.4%
Therapeutic environment	Unclear	53	35.8%
	Moderately clear	80	54.1%
	Clear	15	10.1%
Radiation understanding	Unclear	39	26.3%
	Moderately clear	90	60.8%
	Clear	19	12.9%
Snore	No	78	52.7%
	At least once a month	16	10.8%
	At least once a week	17	11.5%
	1–2 times a week	13	8.8%
	≥3 per week	24	16.2%

### 3.2 Sleep characteristics in patients with differentiated thyroid cancer

At baseline evaluation, the cohort exhibited a mean Pittsburgh Sleep Quality Index (PSQI) score of (6.93 ± 3.65), with 35.8% (53/148) meeting criteria for sleep disturbance (PSQI ≥8). Subsequent assessment demonstrated increased sleep dysfunction prevalence, as evidenced by elevated mean PSQI scores (8.41 ± 3.56) and 52.9% (74/140) classification of impaired sleep quality. At 1-month follow-up, mean scores returned near baseline levels (6.72 ± 3.50), though 32.6% (42/129) maintained clinically significant sleep impairment. Longitudinal psychometric analysis, insomnia severity and sleepiness also revealed dynamic symptom trajectories:Anxiety: 23.6% (T0) → 40.7% (T1) → 14% (T2)Depressive symptoms: 47.3% → 56.4% → 33.3%,Insomnia severity: 41.9% → 61.4% → 46.5%,Daytime somnolence: 32.4% → 42.9% → 31%.

### 3.3 Changes in sleep quality, anxiety and depression scores of patients during three periods of ^131^I treatment

**Figure 2:**
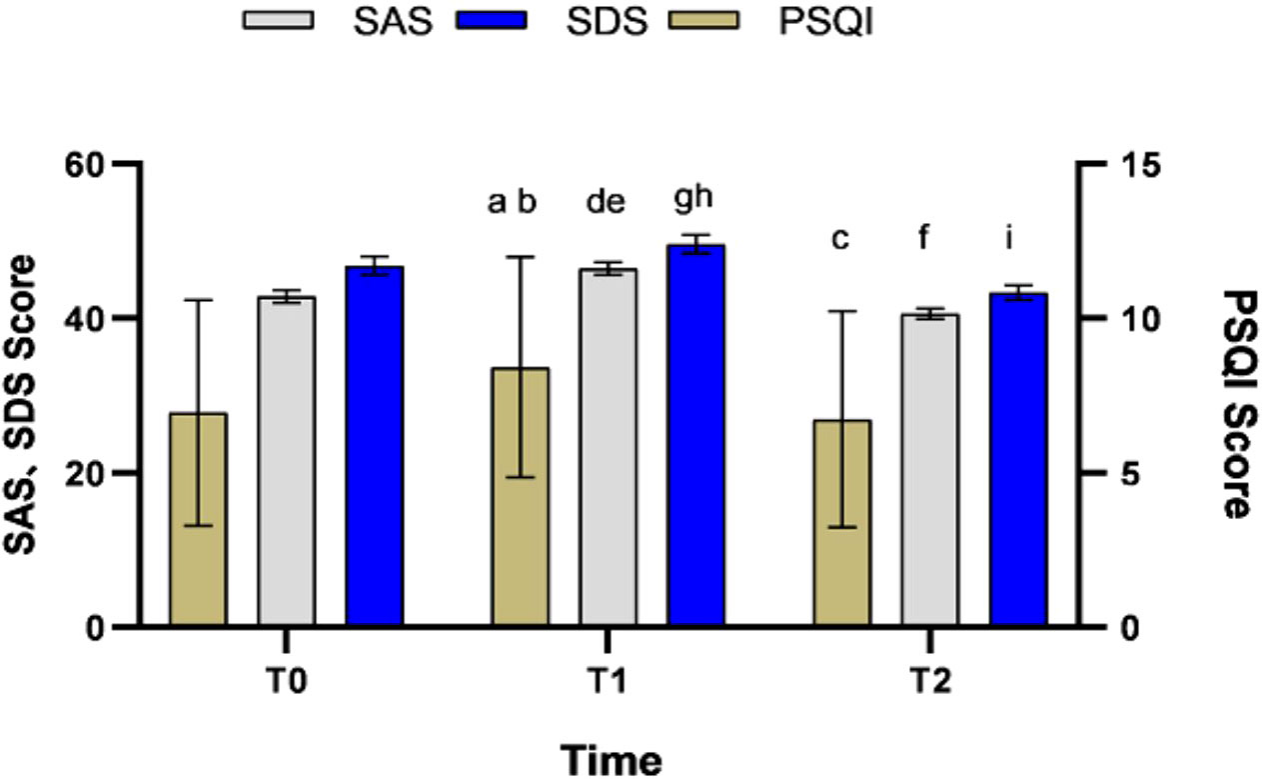
Sleep quality,anxiety and depression indicators were measured longitudinal assessments. ^a,d,g^ Compared with T_0_, P < 0.01; ^b,e,h^ Compared T_1_ with T2, P < 0.01; ^c,f,I^ Compared T_0_ with T_2_, P < 0.05.

With time as the intra-group factor, repeated measurement ANOVA was performed on the sleep quality, anxiety and depression scores of patients in the three time periods. There were statistically significant differences in sleep quality, anxiety and depression scores in the three time periods (P < 0.05), and the pairwise comparison results showed that the pairwise comparison at the three time points was statistically significant (P < 0.05) (**Table 2**). The scores of sleep quality, anxiety and depression showed a trend across the three assessment time (**Figure 2**).

**Table 2:** Comparison of sleep quality, anxiety and depression scores in different periods.

Time	PSQI score	SAS score	SDS score
T_0_	6.93 ± 3.65	42.79 ± 0.83	46.77 ± 1.18
T_1_	8.41 ± 3.56^a^	46.40 ± 0.83^a^	49.58 ± 1.16^a^
T_2_	6.72 ± 3.50^bc^	40.55 ± 0.69^bc^	43.29 ± 0.96^bc^
F	108.650	127.382	106.486
P	<0.001	<0.001	<0.001

a Compared with T_0_; P < 0.01.

b Compared T_1_ with T_2_; P < 0.01.

c Compared T_0_ with T_2_, P < 0.05; T_0_, two weeks prior to receiving ^131^I treatment; T_1_, 24 hours after receiving ^131^I treatment; T_2_, one month after receiving ^131^I treatment; SAS, Self-Rating Anxiety Scale; SDS, Self-Rating Depression Scale; PSQI, Pittsburgh Sleep Quality Index.

### 3.4 Analysis of associated factors of PSQI scores in three time periods

The patients were divided into sleep disorder group and normal sleep group according to PSQI > 7. The chi-square test was used to compare the distribution of sleep disorders under different social demographic characteristics during the three periods of treatment. After treatment, educational background, snoring, time of using mobile phone, taking sleeping pills, work pressure; One month after treatment, snoring, mobile phone use time, taking sleeping pills, work interest, work stress, and radiation were investigated. These were statistically significant in different groups. The results of T-test analysis showed that the anxiety level of patients in the sleep disorder group (PSQI > 7) was higher than that in the normal sleep group (**Tables 3**, **4**).

**Table 3:** Distribution of sleep disorders in DTC patients treated with ^131^I under different social demographic characteristics.

Characteristics		T_0_SD (n=148)	T_1_SD (n=140)	T_2_SD (n=129)
Education	Technical secondary school and below	19(35.8)	27(36.5)	16(38.1)
	Junior college student	11(20.8)	13(17.6)	9(21.4)
	Postgraduate degree	4(7.6)	6(8.2)	4(9.5)
	χ^2^	3.438	11.233	7.727
	P	0.487	0.024	0.102
Snore	No	21(39.6)	30(40.5)	16(38.1)
	At least once a month	3(5.7)	7(9.5)	2(4.8)
	At least once a week	11(20.8)	12(16.2)	9(21.4)
	1–2 times a week	6(11.3)	9(12.2)	5(11.9)
	≥3 per week	12(22.6)	16(21.6)	10(23.8)
	χ^2^	13.588	14.371	11.745
	P	0.009	0.006	0.019
Work interest	High	23(33.3)	33(35.1)	12(28.6)
	Moderate	43(62.3)	57(60.6)	26(61.9)
	Low	3(4.3)	4(4.3)	4(9.5)
	χ^2^	5.244	6.972	7.529
	P	0.073	0.031	0.023
Work stress	Low	26(37.7)	38(40.4)	17(30.9)
	Moderate	41(59.4)	54(57.4)	36(65.5)
	High	2(2.9)	2(2.1)	2(3.6)
	χ^2^	11.011	12.448	14.872
	P	0.004	0.002	0.001
Therapeutic environment	Unclear	23(47.8)	40(42.6)	21(50.0)
	Moderately clear	29(42.0)	44(46.8)	17(40.5)
	Clear	7(10.1)	10(10.7)	4(9.6)
	χ^2^	9.248	5.467	3.858
	P	0.026	0.141	0.277
Radiation understanding	Unclear	25(36.2)	30(31.9)	18(42.9)
	Moderately clear	36(52.2)	50(53.2)	18(42.9)
	Clear	8(11.6)	14(14.9)	6(14.3)
	χ^2^	6.802	4.910	12.183
	P	0.079	0.179	0.007

T_0_, two weeks prior to receiving ^131^I treatment; T_1_, 24 hours after receiving ^131^I treatment; T_2_, one month after receiving ^131^I treatment; SD, Sleep disorder.

**Table 4:** Comparison of anxiety and depression scores between sleep disorder group and normal sleep group.

Measure and time point	Sleep disorder group (n=53)	Normal sleep group (n=95)	t	P
Self-Rating Anxiety Scale (SAS) score				
Two weeks before RAI therapy (T0)	46.92 ± 9.41	40.01 ± 8.19	4.665	<0.001
One day after RAI therapy (T1)	50.22 ± 8.81	41.89 ± 7.82	5.883	<0.001
One month after RAI therapy (T2)	45.64 ± 6.95	38.09 ± 7.03	5.734	<0.001
Self-Rating Depression Scale (SDS) score				
Two weeks before RAI therapy (T0)	51.79 ± 12.21	43.47 ± 12.89	3.835	<0.001
One day after RAI therapy (T1)	54.11 ± 11.66	44.68 ± 12.84	4.553	<0.001
One month after RAI therapy (T2)	49.29 ± 9.74	40.40 ± 10.34	4.658	<0.001

### 3.5 Logistic regression analysis of related factors in different stages of sleep disorders

The results of the logistic regression analysis of sleep disorders before and after treatment indicated that the anxiety level of the patients, snoring, and work pressure were the risk factors for sleep disorders before treatment; snoring, the anxiety level, and educational attainment were the risk factors for sleep disorders after the end of treatment; the anxiety level and snoring were the risk factors for sleep disorders after treatment (**Tables 5**–**7**).

**Table 5:** Logistic regression analysis of factors related to sleep quality before treatment.

					95%CI	
Variate	β	SE	P	OR	Min	Max
Constant term	-5.365	1.687	0.001	0.005		
Anxiety	0.07	0.035	0.046	1.072	1.001	1.148
Snore (Compared with no snoring)						
At least once a week	1.632	0.721	0.024	5.112	1.245	20.989
Working pressure (as opposed to Low )						
High	0.932	0.458	0.042	2.541	1.036	6.228

**Table 6:** Logistic regression analysis of sleep quality related factors 24 hours after treatment.

					95%CI	
Variate	β	SE	P	OR	Min	Max
Constant term	-6.441	1.669	0.000	0.002		
Anxiety	0.124	0.043	0.004	1.132	1.040	1.232
Snore (Compared with no snoring)						
1–2 times a week	1.472	1.024	0.026	9.736	1.308	70.477
≥3 per week	1.472	0.722	0.041	4.359	1.059	17.936
Education(Compared with Technical secondary school )						
Undergraduate	1.139	0.568	0.045	3.123	1.026	9.505

**Table 7:** Logistic regression analysis of factors related to sleep quality one month after treatment.

					95%CI	
Variate	β	SE	P	OR	Min	Max
Constant term	-9.178	2.091	0.000	0.000		
Anxiety	0.210	0.069	0.002	1.233	1.078	1.411
Snore (Compared with no snoring)						
1–2 times a week	1.693	0.838	0.043	5.438	1.053	28.082

## 4 Discussion

Differentiated thyroid cancer (DTC) is the most common subtype of thyroid cancer, with radioactive iodine (^131^I) therapy serving as a cornerstone of its management. This prospective longitudinal study revealed significant insights into the sleep quality and psychological well-being of patients with DTC undergoing ^131^I therapy. We observed dynamic changes in sleep and psychological symptoms across the pre-, peri-, and post-treatment phases. Notably, the incidence of sleep disorders, anxiety, and depressive symptoms exhibited a non-linear pattern, peaking during or immediately after treatment before showing partial recovery at one-month follow-up. Despite the growing recognition of psychology and sleep adverse effects, the temporal patterns and underlying mechanisms remain poorly understood, highlighting the need for further research to optimize patient care and therapeutic outcomes.

Our findings confirm the high prevalence of poor sleep quality, anxiety, and depression among DTC patients, which is consistent with prior longitudinal studies highlighting the significant impact of ¹³¹I therapy on mental health (20, 21). The reported prevalence rates of anxiety (≈30%) and depression (≈20%) in DTC populations further contextualize the substantial psychological burden observed in our cohort (22).The persistent nature of these challenges highlights a critical gap in supportive care, necessitating targeted interventions throughout the treatment trajectory.

The longitudinal design of our study allowed us to delineate the temporal pattern of these symptoms. The peak in sleep disturbances, anxiety, and depression during the peri-therapeutic phase coincides with the acute period of ¹³¹I administration. This timing is consistent with the acute stress model, encompassing concerns over treatment efficacy, side effects, and required isolation (23, 24).The subsequent decline in symptoms at the one-month follow-up parallels findings from other studies (25). This non-linear trajectory challenges the notion of a simple, linear psychological progression (26), and underscores the complex interplay of treatment-related, psychological, and social factors over time.

Our observation of a significant decline in sleep quality immediately following RAI therapy (peri-therapeutic phase) aligns with the findings of He et al., who similarly reported acute sleep deterioration during this period (27). In our cohort, anxiety, snoring, educational level, and work/study-related stress were significantly associated with an increased risk of sleep disturbances. The identification of phase-specific factors, such as pre-treatment work-related stress (28) and peri-treatment anxiety (29), suggests that a dynamic, phase-specific care model may be beneficial. For example, proactive stress management and sleep hygiene education before treatment may mitigate baseline risk.

During the acute treatment phase, our data support the critical role of intensified psychological support and sleep monitoring to address symptom peaks. The potential for sleep disturbances to persist in a subset of patients, as supported by Koo et al. (13) and observed in our cohort, reinforces the recommendation from survivorship studies that sleep quality should be routinely assessed in long-term follow-up, even in low-risk patients (30, 31). Notably, snoring may serve as a marker for potential sleep apnea, emphasizing the importance of comprehensive sleep assessments (32).

### 4.1 Limitations and future directions

Several limitations of this study should be considered. First, the observational design precludes causal inference; the associations reported may be influenced by unmeasured confounders. Second, although longitudinal, data on several clinically relevant variables (e.g., radioiodine dose, tumor stage, use of recombinant TSH) were not consistently available for formal adjustment. Third, the sample was from a single center, which may affect the generalizability of the findings. Future research should include multi-center studies with longer follow-up and more comprehensive biomarker assessments to confirm these patterns and investigate underlying biological mechanisms, such as the role of inflammatory pathways or hormonal changes, to inform the development of more effective interventions.

### 4.2 Conclusion

In conclusion, this study provides valuable insights into the sleep quality and psychological well-being of DTC patients undergoing 131I therapy. The high prevalence of sleep disturbances, anxiety, and depression, along with their dynamic trajectories and interrelationships, highlights the urgent need for a holistic and personalized approach to care. By addressing the identified risk factors and implementing targeted interventions, healthcare providers can effectively mitigate adverse psychological and physiological outcomes and enhance overall treatment efficacy. In clinical practice, it is indispensable to diligently monitor the mental health status of DTC patients both before and after treatment and offer tailored psychological support services throughout the entire diagnosis and treatment process. Integrating psychological counseling into the clinical workflow can significantly improve patients’ quality of life and treatment outcomes.

## Data Availability

The raw data supporting the conclusions of this article will be made available by the authors, without undue reservation.
